# Biological Effect Evaluation of Glutathione-Responsive Cyclodextrin-Based Nanosponges: 2D and 3D Studies

**DOI:** 10.3390/molecules25122775

**Published:** 2020-06-16

**Authors:** Monica Argenziano, Federica Foglietta, Roberto Canaparo, Rita Spagnolo, Carlo Della Pepa, Fabrizio Caldera, Francesco Trotta, Loredana Serpe, Roberta Cavalli

**Affiliations:** 1 Department of Drug Science and Technology, University of Torino, Via Pietro Giuria 9, 10125 Torino, Italy; monica.argenziano@unito.it (M.A.); federica.foglietta@unito.it (F.F.); roberto.canaparo@unito.it (R.C.); rita.spagnolo@unito.it (R.S.); carlo.dellapepa@unito.it (C.D.P.); loredana.serpe@unito.it (L.S.); 2 Department of Chemistry, University of Torino, Via Pietro Giuria 7, 10125 Torino, Italy; fabrizio.caldera@unito.it (F.C.); francesco.trotta@unito.it (F.T.)

**Keywords:** nanosponges, β-cyclodextrin, glutathione, cancer, multicellular spheroids

## Abstract

This study aims to evaluate the bioeffects of glutathione-responsive β-cyclodextrin-based nanosponges (GSH-NSs) on two- (2D) and three-dimensional (3D) cell cultures. The bioeffects of two types of GSH-NS formulations, with low (GSH-NS B) and high (GSH-NS D) disulfide-bond content, were evaluated on 2D colorectal (HCT116 and HT-29) and prostatic (DU-145 and PC3) cancer cell cultures. In particular, the cellular uptake of GSH-NS was evaluated, as their effects on cell growth, mitochondrial activity, membrane integrity, cell cycle distribution, mRNA expression, and reactive oxygen species production. The effect of GSH-NSs on cell growth was also evaluated on multicellular spheroids (MCS) and a comparison of the GSH-NS cell growth inhibitory activity, in terms of inhibition concentration (IC)_50_ values, was performed between 2D and 3D cell cultures. A significant decrease in 2D cell growth was observed at high GSH-NS concentrations, with the formulation with a low disulfide-bond content, GSH-NS B, being more cytotoxic than the formulation with a high disulfide-bond content, GSH-NS D. The cell growth decrease induced by GSH-NS was owing to G_1_ cell cycle arrest. Moreover, a significant down-regulation of mRNA expression of the cyclin genes *CDK1*, *CDK2*, and *CDK4* and up-regulation of mRNA expression of the cyclin inhibitor genes *CDKN1A* and *CDKN2A* were observed. On the other hand, a significant decrease in MCS growth was also observed at high GSH-NS concentrations, but not influenced by the nanosponge disulfide-bond content, with the MCS IC_50_ values being significantly higher than those obtained on 2D cell cultures. GSH-NSs are suitable nanocarries as they provoke limited cellular effects, as cell cycle arrest only occurred at concentrations significantly higher than those used for drug delivery.

## 1. Introduction

The ideal nanoparticle-based drug delivery system assures the safe delivery and selective action of a drug to a target site. Indeed, nanomaterials can add further functionality to the conjugated/loaded drug and, taking advantage of their unique size, are able to play a crucial therapeutic role. This has triggered an increased interest in nanopharmaceuticals [1,2] and the development of a wide range of nanoparticle systems, such as liposomes, nanoparticles, micelles, dendrimers, and nanotubes [3,4,5]. However, only a few nanoparticle-based systems have been FDA-approved for cancer therapy to date [6,7]. Although nanoscience in drug development is in its early stages, the fusion of engineered nanomaterials and nanopharmaceutical research is paving the way for the development of stimuli-responsive drug delivery systems, especially in cancer treatment. Interestingly, several chemically modified polymers [8,9] and cross-linked cyclodextrin-based polymers have been proposed to obtain compounds responsive to the external environment [10]. In this regard, cyclodextrin-based nanosponges are of particular interest [11,12].

Nanosponges are hyper-cross-linked cyclodextrin polymers generally obtained from α, β, and γ cyclodextrins, containing suitable amounts of linear dextrin cross-linked with a proper cross-linking agent. A cage-like structure is obtained via the cross-linking of cyclodextrins, thus creating nanochannels in the polymer matrix that can be modulated employing different types of cross-linking agents and/or varying the amount used [13,14]. It is worth noting that active carbonyl compounds, like carbonyl diimidazole, diphenyl carbonate, and organic dianhydrides such as pyromellitic dianhydride, can be used as cross-linker in the preparation of nanosponges [15,16].

Nanosponges offer several features, such as sustained and controlled release, improvement of aqueous solubility, bioavailability, and stability of the hosted molecules, which could be advantageously exploited for drug delivery [15,17,18]. Indeed, previous research highlighted the capability of nanosponges to encapsulate different active molecules and magnify their activity in either in vitro or in vivo studies [15,19,20]. In particular, several anticancer drugs such as doxorubicin, paclitaxel, and camptothecin have been efficiently incorporated in cyclodextrin-based nanosponges, showing an improved antitumor effect [21,22,23,24].

Interestingly, nanosponge-based drug delivery systems can be tuned to form ‘stimuli-responsive’ nanocarriers that modify their structure in response to external changes, such as pH or redox potential [25,26,27]. Therefore, glutathione (GSH)-responsive nanocarriers have been developed for targeted intracellular anticancer drug release [28], as the GSH tripeptide has a higher intracellular than extracellular concentration [29]. Several intracellular compartments, such as cytosol, mitochondria, and the cell nucleus, contain higher GSH concentrations than do extracellular fluids and circulation. Moreover, GSH intracellular concentration is further increased in cancer cells and, above all, in chemoresistant cells [30]. Oxidative stress has long been implicated in cancer development and progression [31]. An increase in reactive oxygen species (ROS) usually induces a cell adaptive response and the compensatory up-regulation of antioxidant systems to restore redox homeostasis; GSH/GSH disulfide is the major redox combination in mammalian cells [32]. Moreover, many primary tumors have high levels of overexpression of antioxidant enzymes [33,34].

Trotta et al. have developed a next generation of nanosponges that are bioresponsive to GSH external concentration [35]. This behavior may be an ideal trigger for rapid nanocarrier destabilization inside cells, leading to efficient intracellular drug release through disulfide-bond cleavage [35]. Indeed, the disulfide bridge remains stable in extracellular fluids for long periods before being reduced upon internalization in the cytosol, having a higher GSH concentration, thus improving drug bioavailability [36]. As depleting endogenous antioxidants, like GSH, make cancer cells more chemosensitive, this reduction-sensitive nanosystem is further suited to anticancer therapy owing to its ability to enhance the anticancer activity of such drugs. Previously, the encapsulation of doxorubicin and strigolactone analogues in GSH-responsive nanosponges was in vitro and in vivo evaluated. In vitro release kinetics studies from GSH-NS revealed a GSH concentration-based drug release profile over time. Moreover, the GSH-NSs were able to release the payload as a function of in-cell GSH concentration [37,38]. This behavior might favor the selectively controlled release in target cancer cells and enhance the cytotoxic effect. Indeed, both of the compounds loaded in GSH-NS were more effective in inhibiting the cell viability than the corresponding free drugs, particularly in cancer cells presenting a higher GSH content. In addition, a greater reduction of prostate cancer growth was observed for doxorubicin incorporated in GSH-NS compared with the free drug in xenograft mice models [37].

The fact that GSH-NS may well represent an efficient stimuli-responsive drug delivery system for anticancer drugs prompted in-depth study into their biological effect per se on cell growth reported herein. As preliminary cellular evaluations of nanocarriers are usually carried out on 2D cell cultures, previously, the effects of cyclodextrin-based nanosponges have been widely tested in 2D cell monolayer cultures. However, 3D cell cultures, such as multicellular spheroids (MCS), have various in vivo tissue characteristics including the production of an extracellular matrix [39,40]. This study reports a series of experiments carried out to evaluate the bioeffects of GSH-NS containing two different amounts of disulfide bridges either on 2D cell cultures or on 3D cell cultures of human cancer cells, differing in cancer type and intracellular GSH level, namely, human colorectal, HCT116 and HT-29, and human prostatic, DU145 and PC-3, cancer cell lines.

## 2. Results

### 2.1. Characterization of Glutathione Responsive β-cyclodextrin-Based Nanosponges

GSH-NSs have a size of about 200 nm and a negative surface charge, in agreement with our previous papers [35,37,38]. Table 1 reports the average diameters, polydispersity indices, and zeta potentials of blank and 6-coumarin loaded GSH-NS type B and D. The different content of disulfide bridges in the two type of GSH-NS (B and D) did not affect their physico-chemical characteristics. Fluorescent labeling did not significantly alter the values of these parameters. Transmission electron microscopy (TEM) analysis of GSH-NS shows the spherical shape and smooth surfaces of the nanosponges.

### 2.2. D Cell Culture Cytotoxicity of Glutathione Responsive β-cyclodextrin-Based Nanosponges

**Figure 1:**
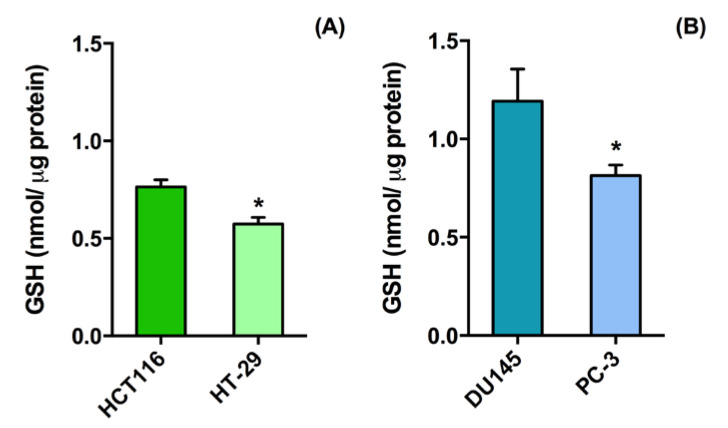
Intracellular glutathione-responsive β-cyclodextrin (GSH) level according to cell type. The reduced glutathione content of human colorectal cancer cell lines, HCT 116 and HT-29 (**A**), and human prostatic carcinoma cell lines, DU145 and PC-3 (**B**), was measured at a basal level, that is, in untreated cells, and was expressed as nmol/µg protein. The results are mean values ± SD of three independent experiments performed in triplicate. Statistically significant difference between cell lines: * *p* < 0.05.

The basal level of reduced glutathione was measured in each of the different cell lines, which were grouped according to cancer type. The results show that HT-29 (Figure 1A) and PC-3 cells (Figure 1B) display significantly lower GSH levels than those in HCT116 (Figure 1A) and DU145 cells (Figure 1B). Dose-response curves were then performed by exposing human colorectal cancer, HCT116 and HT-29, and human prostatic carcinoma, DU145 and PC-3, cell monolayers to various concentrations (0.5, 1.0, 2.0, and 3.0 mg/mL) of the two types of GSH-NSs with increasing disulfide bridge content (GSH-NS B and D), for 24, 48, and 72 h. Table 2 reports the IC_1_ and IC_50_ values of GSH-NS, which were determined at 24, 48, and 72 h of exposure.

**Figure 2:**
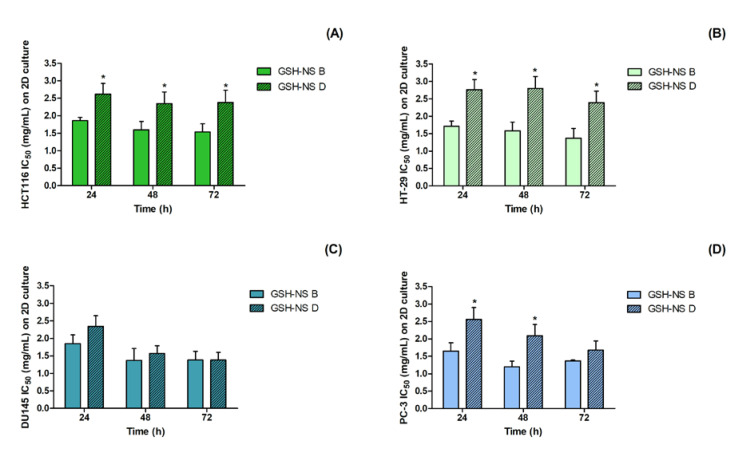
Cytotoxicity of glutathione responsive β-cyclodextrin-based nanosponges in 2D cell cultures. HCT116 (**A**), HT-29 (**B**), DU145 (**C**), and PC-3 (**D**) cells were incubated with GSH-NS B and GSH-NS D at different concentrations (0.5, 1.0, 2.0, 3.0 mg/mL) for 24, 48, and 72 h. Cell proliferation was evaluated by WST-1 assay and the values of the concentration required for a 50% cell growth inhibition (IC_50_) were determined by the proliferation curves obtained using the CalcuSyn 2.11 software (Biosoft, Cambridge, UK). The results are mean values ± St.Dev of three independent experiments, replicated eight times for each condition. Statistically significant difference between nanosponge formulations: * *p* < 0.05.

Figure 2 reports GSH-NS IC_50_ values according to disulfide-bond content and exposure time. These data highlight the remarkable cytotoxicity difference between the two nanosponge formulations in colorectal cancer cell lines, as lower IC_50_ values were observed for the nanosponge formulation with the lower disulfide bridge content (GSH-NS B) at 24, 48, and 72 h (Figure 2A,B). In prostatic cancer cell lines, no significant differences in IC_50_ values were detected for the two nanosponge formulations in DU145 cells (Figure 2C), which were characterized by the highest GSH cell content (Figure 1). Meanwhile, in PC-3 cells, a significant cytotoxicity difference was observed between the two nanosponge formulations, as the IC_50_ values determined by the GSH-NS B formulation were lower compared with what was observed in colorectal cancer cells, even if only at 24 and 48 h (Figure 2D).

Therefore, the nanosponge formulation with the lower disulfide-bond content, GSH-NS B, was found to be more cytotoxic than the nanosponge formulation with the higher disulfide-bond content, GSH-NS D, in all of the cell lines, with the exception of DU145 cell line (Table 2 and Figure 2). Moreover, lower GSH-NS D IC_50_ values were observed on DU145 and PC-3 cells than on HCT116 and HT-29 cells at 48 and 72 h of exposure (Table 2 and Figure 2), suggesting that the nanosponge formulation with the higher disulfide bridge concentration, GSH-NS D, has a higher cytotoxic effect in prostatic cancer cells than in colorectal cancer cells.

### 2.3. Glutathione Responsive β-cyclodextrin-Based Nanosponge Cellular Uptake on 2D Cell Cultures

**Figure 3:**
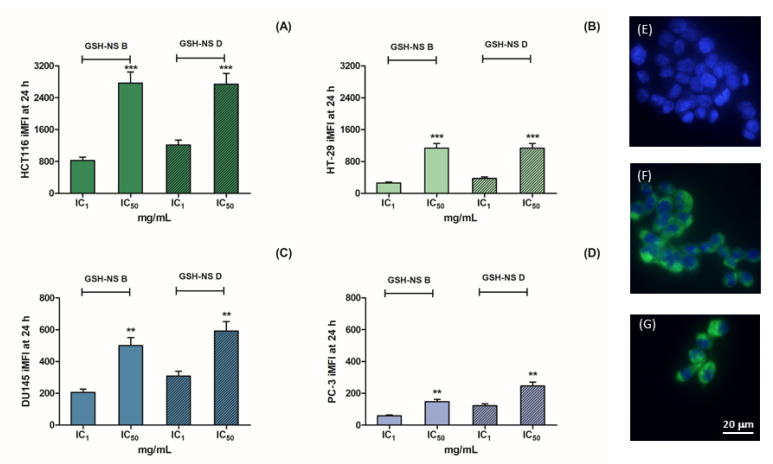
Fluorescent glutathione responsive β-cyclodextrin-based nanosponge cellular uptake. HCT116 (**A**), HT-29 (**B**), DU145 (**C**), and PC-3 (**D**) cells were exposed to the respective IC_1_ and IC_50_ of 6-coumarin loaded GSH-NS B and 6-coumarin loaded GSH-NS D for 24 h and analyzed by flow cytometry. Cellular uptake was expressed as integrated mean fluorescence intensity (iMFI). Representative fluorescence images of HCT116 untreated cells (**E**), HCT116 cells exposed to 6-coumarin loaded GSH-NS B IC_50_ (**F**), and 6-coumarin loaded GSH-NS D IC_50_ (**G**) for 24 h using 4′,6-diamidino-2-phenylindole (DAPI) (blue) as nuclear counterstain (63x magnification). Statistically significant difference between IC_1_ and IC_50_: ** *p* < 0.01; *** *p* < 0.001.

The cellular uptake of non-cytotoxic (IC_1_) and cytotoxic (IC_50_) concentrations of fluorescent GSH-NS was analyzed by flow cytometry and fluorescence microscope imaging after 24 h exposure. HCT116, HT-29, DU145, and PC-3 cell monolayers were exposed to 6-coumarin loaded GSH-NS B or 6-coumarin loaded GSH-NS D for 24 h. Significant dose-dependent differences in nanosponge cellular uptake were observed (Figure 3A–D). Interestingly, higher nanosponge cellular uptake was observed in colorectal cancer cells (Figure 3A,B) than in prostatic cancer cells (Figure 3C,D). In particular, the highest nanosponge cellular uptake was observed in HCT116 cells (Figure 3A) and the lowest in PC-3 cells (Figure 3D). As IC_50_ values at 24 h of incubation in colorectal and prostatic cell line were very similar (Table 2), the lower fluorescent GSH-NS intracellular uptake in prostatic cells suggests that they are more highly sensitive to the nanosponge cytotoxic effect than the colorectal cancer cell lines. In particular, Figure 3E–G show images of untreated HCT116 cells with nuclear counterstaining (Figure 3E) and after 24 h exposure to 6-coumarin loaded GSH-NS B (Figure 3F) or 6-coumarin loaded GSH-NS D (Figure 3G).

### 2.4. The Effect of Glutathione Responsive β-cyclodextrin-Based Nanosponges on Cell Death and Cell Cycle

As an increase in the number of dead or plasma membrane-damaged cells results in an increase in lactate dehydrogenase (LDH) in the culture supernatant, we measured the LDH leakage of HCT116, HT-29, DU145, and PC-3 cells after 24, 48, and 72 h of incubation with an experimental medium containing different GSH-NS B or GSH-NS D concentrations (0.5, 1.0, 2.0, and 3.0 mg/mL). No significant increase in LDH leakage percentage over untreated control cells was observed under any of the test conditions (data not shown). The lack of apparent plasma membrane-damaged cells in the LDH assay would appear to contrast significantly with the decrease in cell growth observed by WST-1 cell proliferation assay (Table 2 and Figure 2), which was performed at the same incubation times with the same GSH-NS concentrations. This finding prompted us to investigate the cell cycle using flow cytometry to assess any arrest of cell cycle progression.

**Figure 4:**
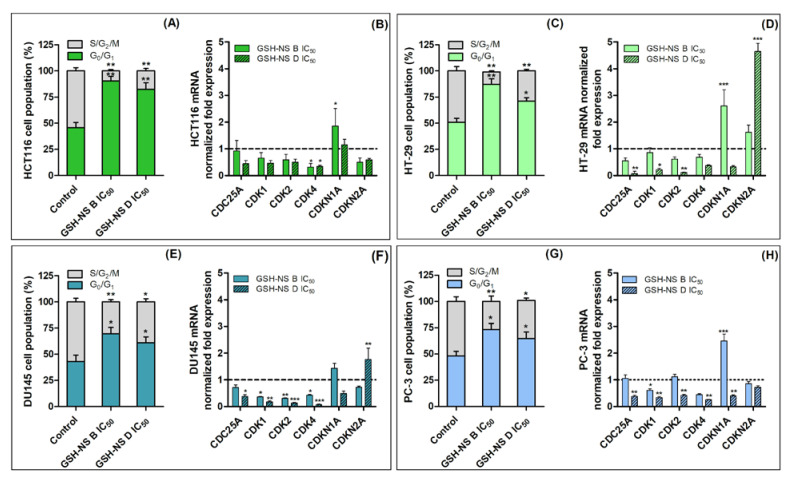
Glutathione responsive β-cyclodextrin-based nanosponge effect on cell cycle and mRNA expression. HCT116 (**A**,**B**), HT-29 (**C**,**D**), DU145 (**E**,**F**), and PC-3 (**G**,**H**) cells were exposed to the respective GSH-NS B or GSH-NS D IC_50_ for 24 h. Cell cycle distribution was analyzed by flow cytometry and the data were expressed as a percentage of cells in the different phases of the cell cycle (**A**,**C**,**E**,**G**). *RRN18S* (ribosomal RNA 18S) was used for the mRNA gene expression analysis as a reference gene to normalize the data and the nanosponge-induced alterations in mRNA levels were compared with those of the controls, that is, untreated cells, fixed at 1 and shown by the dotted line. Statistically significant difference versus control: * *p* < 0.05; ** *p* < 0.01; *** *p* < 0.001.

We then performed cell cycle analyses on the IC_50_ values of each cell line after 24 h of GSH-NS B or GSH-NS D incubation (Table 2). A significant increase in cell percentages in the G_0_/G_1_ phase and a significant decrease in S/G_2_/M cell percentages were observed across the entire cell population after exposure at the respective GSH-NS B or GSH-NS D IC_50_ values (Figure 4). Moreover, the sub-G_0_/G_1_ peak was absent in all cell lines. This suggests that the observed decrease in cell proliferation (Table 2 and Figure 2) was the result of alterations of cell cycle progression owing to a block in the G_0_/G_1_ phase. Furthermore, the G_0_/G_1_ phase cell population percentage was higher in both colorectal cancer (Figure 4A,B) and prostatic cancer (Figure 4C,D) cells after incubation with the lower disulfide-bond content nanosponge (GSH-NS B).

Thus, to confirm these data, an analysis of mRNA expression of the different cyclin-dependent kinases (CDK) was performed, as they participate in cell cycle regulation, especially during the G_1_ to S phase transition. It was observed that *CDK1*, *CDK2*, and *CDK4* mRNA expression was down-regulated in almost all cell lines, as compared with untreated cells (Figure 4B,D,F,H). Interestingly, the mRNA expression of the CDK activator, *CDC25A*, was either unaffected or down-regulated, compared with untreated cells, whereas the mRNA expression of the CDK inhibitors, *CDKN1A* and *CDKN2A*, was up-regulated compared with untreated cells (Figure 4B,D,F,H). No significant differences in the extent of cell cycle arrest were observed between the two nanosponge formulations. However, the formulation with the higher disulfide-bond content, GSH-NS D, seemed to induce a higher down-regulation in CDK mRNA and higher up-regulation in *CDKN2A* mRNA in HT-29 and DU145 cells, whereas GSH-NS B induced a higher *CDKN1A* mRNA expression in HCT116 and PC-3 cells. Furthermore, no significant differences were observed in terms of the extent of cell cycle arrest and gene expressions between cell lines with higher (Figure 4A–F) and lower GSH content (Figure 4C,D,G,H).

### 2.5. The Effect of Glutathione Responsive β-cyclodextrin-Based Nanosponges on Reactive Oxygen Species Production

**Figure 5:**
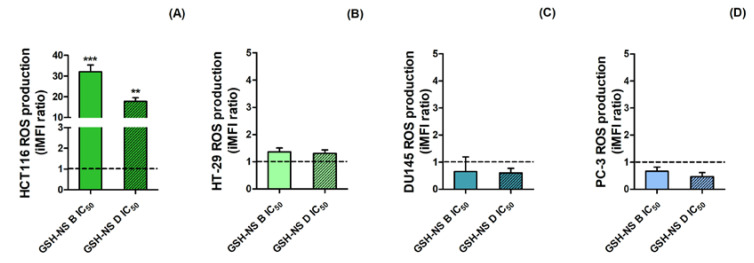
Glutathione responsive β-cyclodextrin-based nanosponge reactive oxygen species (ROS) production. HCT116 (**A**), HT-29 (**B**), DU145 (**C**), and PC-3 (**D**) cells were treated with GSH-NS B or GSH-NS D at the respective IC_50_ for 24 h. ROS levels, detected by dichlorofluorescein-diacetate (DCFH-DA) assay by flow cytometry, were expressed as the integrated median fluorescence intensity (iMFI) ratio and nanosponge-induced ROS levels were compared to those of control, that is, untreated cells, fixed at 1 and shown by the dotted line. Statistically significant difference versus untreated cells: ** *p* < 0.01; *** *p* < 0.001.

After exposure to the respective GSH-NS B and GSH-NS D IC_50_ values at 24 h, the dichlorofluorescein-diacetate (DCFH-DA) assay did not indicate a significant intracellular ROS increase at 1, 12 (data not shown), and 24 h (Figure 5) in all cell lines considered, except for HCT116 cells, where a significant increase in intracellular ROS was observed at 24 h (Figure 5A) after both GSH-NS formulations incubation (Figure 5A).

### 2.6. Glutathione Responsive β-cyclodextrin-Based Nanosponge Cytotoxicity in Three-Dimensional Cell Cultures

**Figure 6:**
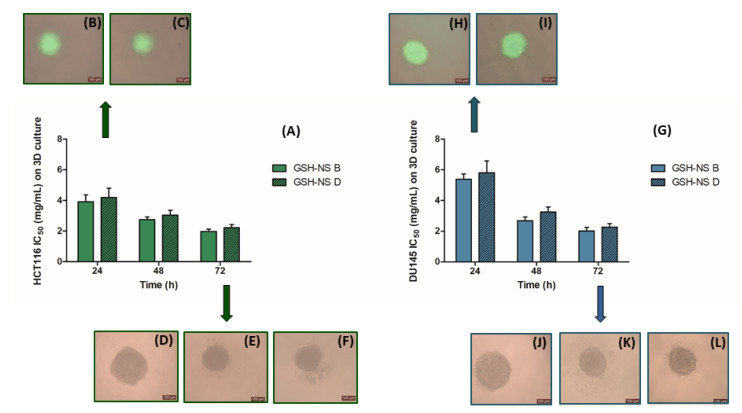
Glutathione responsive β-cyclodextrin-based nanosponge cytotoxicity and uptake on 3D culture. HCT116 (**A**) and DU145 (**G**) multicellular spheroids (MCSs) were incubated with GSH-NS B and GSH-NS D at different concentrations (0.5, 2.0, 4.0, and 6.0 mg/mL) and MCS volume was measured after 24, 48, and 72 h. Representative images at 24 h of HCT116 and DU145 MCS uptake of 6-coumarin loaded GSH-NS B IC_50_ (**B**,**H**, respectively) and of 6-coumarin loaded GSH-NS D IC_50_ (**C**,**I**, respectively). Representative phase contrast images at 72 h of HCT116 MCS: untreated (**D**), treated with GSH-NS B IC_50_ (**E**) and GSH-NS D IC_50_ (**F**). Representative phase contrast images at 72 h of DU145 MCS: untreated (**J**), treated with GSH-NS B IC_50_ (**K**), and GSH-NS B IC_50_ (**L**). Images are at 10× magnification.

The next step was the analysis of GSH-NS cytotoxicity in the colorectal and prostatic cancer cell lines with the highest GSH basal level, HCT116 and DU145, which were three-dimensionally cultured as multicellular spheroids (MCSs), cellular aggregates organized in a specific cell-to-cell and cell–matrix interaction, closer to in vivo features [41]. Dose response curves were obtained by exposing HCT116 and DU145 on 3D model to different concentrations (0.5, 2.0, 4.0, and 6.0 mg/mL) of GSH-NS B and GSH-NS D, for 24, 48, and 72 h to obtain the respective IC_1_ and IC_50_ values (Table 3 and Figure 6A,G). The MCS uptake of non-cytotoxic (IC_1_) and cytotoxic (IC_50_) concentrations of fluorescent GSH-NS was confirmed by fluorescence microscope imaging after 24 h of exposure (Figure 6B,C,H,I).

GSH-NS B and D gave similar IC_50_ values in both cell lines (Table 3, Figure 6A,G) and across all incubation times, whereas GSH-NS B gave a higher cytotoxic effect in HCT116 cells than GSH-NS D in the monolayer cultures (Table 2 and Figure 2A). Interestingly, the nanosponge cytotoxic effect was time dependent in both cell lines (Figure 6A,G). This also differs from the results obtained in the monolayer cultures (Figure 2A,C). Moreover, significantly higher GSH-NS B and GSH-NS D IC_50_ values at 24 h were observed in DU145 cells than in HCT116 cells (Figure 6G), suggesting a lower cytotoxic effect of the GSH-NS at 24 h in DU145 cells than in HCT116 cells. These results differ from those observed in monolayer cultures, where the nanosponge formulation with the higher disulfide-bond content, GSH-NS D, was more cytotoxic in DU145 cells than in HCT116 (Figure 2A,C). Figure 6 shows images of 72 h untreated HCT116 MCS (10.51 ± 1.74 μm^3^_,_ panel D) and DU145 MCS (11.66 ± 0.92 μm^3^, panel J), which are compared to GSH-NS B and GSH-NS D treated HCT116 MCS (4.03 ± 0.09 μm^3^, panel E and 4.55 ± 0.77 μm^3^, panel F, respectively) (*p* < 0.05) and DU145 MCS (4.63 ± 0.27 μm^3^, panel K and 5.12 ± 0.60 μm^3^, panel L, respectively) (*p* < 0.05).

## 3. Discussion

Understanding the effect that nanoparticles have on cells is crucial to predict their in vivo toxicity and avoid any undesirable nanoparticle activities. Although there are numerous in vitro cytotoxicity assays that can be applied for the general screening of nanoparticles [42,43], it is of vital importance that the research covers nanoparticle cytotoxicity itself. In this contest the use of strictly controlled in vitro experimental conditions can ensure that the measured effect is the result of nanoparticle toxicity and not unstable culturing conditions [44]. Moreover, up to nowadays, there has been no single analysis able to provide sufficient information to correlate the biomaterial chemistry and surface with biological response [45]. Herein, we investigated the in vitro biological effects of a stimuli-responsive nanosystem, that is, glutathione responsive β-cyclodextrin-based nanosponges (GSH-NS), in various cancer cell lines, characterized by their GSH basal content, as this nanosystem is designed to be a GSH responsive anticancer drug carrier.

GSH plays a key role in cellular defense against oxidative stress [46] and its increased redox capacity in cancer cells is well-known [34,47]. Consequently, GSH has been recognized to be an ideal intracellular trigger for selective drug delivery by responsive nanocarriers, as many compounds exert their therapeutic effects only inside cells. As disulfide chemistry is particularly versatile, a wide range of GSH-responsive nano-vehicles, such as micelles, nanoparticles, and nanogels, have been recently developed [28]. Among them, glutathione responsive β-cyclodextrin-based nanosponges incorporate high payload and provide controlled drug release over time, with the further advantage of triggered intracellular drug delivery in response to cell GSH content. In addition, GSH-NSs are able to protect degradable drugs from the external environment. It is foreseen that β cyclodextrin-based nanosponges will have a significant positive impact on anticancer therapeutic scenarios [13,24,37,38]. Taking into account the promising results concerning the efficacy of GSH-NSs as an anticancer drug delivery system [37,38], the biological safety of the nanosponge itself is a critical parameter for their future clinical application.

β-cyclodextrin toxicology has been evaluated in in vitro and in vivo studies that have reported it as non-toxic and well tolerated even at very high doses [48]. Previous in vitro studies showed no signs of cytotoxicity after cell exposure to unloaded nanosponges in the 10–100 μg/mL concentration range used for the delivery of therapeutic drugs [23,49,50]. In addition, in vivo experiments have shown that β-cyclodextrin-based nanosponges prepared with pyromellitic dianhydride as a cross-linking agent have been orally administered to rats without showing any toxic side effects at selected doses in an acute and repeated dose toxicity study [51]. Previously, GSH-NSs have been investigated as doxorubicin carrier. No acute cardiotoxic effects were observed in mice after the in vivo administration of doxorubicin-loaded GSH-NS [37]. Recently, the hepatotoxicity of this nanoformulation was investigated either in vitro on human HepG2 cell line or ex vivo on rat precision-cut liver slices (PCLSs), where a good nanosponge safety profile was demonstrated, showing a comparable hepatotoxicity to that of free doxorubicin [52].

As no reports have been published on the effects at a cellular level of GSH-NS as such, it was decided to study the effect of GSH-NS per se on HCT116, HT-29, DU145, and PC-3 cancer cell lines with various GSH content in a concentration range that is about fifty times higher than that used in the above mentioned studies to ensure the use of cytotoxic concentrations. HCT116 and DU145 cells showed the highest GSH values in colorectal and prostatic cancer cell lines, respectively; previous research studies have shown that DU145 cells have the highest GSH content [53]. Non-toxic (IC_1_) and cytotoxic (IC_50_) GSH-NS concentrations were determined by a 2D cell assay, which measured mitochondrial activity. A decrease in cell growth with significantly different IC_1_ or IC_50_ values was observed when the two nanosponge formulations were compared in all cell lines, except in DU145 cell line, where no statically significant difference was observed.

DU145 cell line was the most sensitive to the GSH-NS D cytotoxic effect among all cell lines tested. Notably, DU145 cells are more resistant to electrophilic toxicity than other cells owing to their high levels of redox-sensitive transcription factor, nuclear factor erythroid 2-related factor-2 (Nrf2), which activates cytoprotective pathways against oxidative injury, such as GSH synthesis [54,55]. As this nanosystem has the ability to disrupt itself in the presence of GSH, we can hypothesize that it is the high GSH content in DU145 cells that allows GSH-NS to exert their cytotoxic effect, whatever the disulfide-bond concentration. Further studies are needed to investigate whether agents able to modulate intracellular GSH, such as *N*-acetyl cysteine or buthionine sulfoximine [56] could affect nanosponge intracellular drug release and cytotoxicity.

Our study shows that colorectal cancer cells, in particular HCT116 cells, have the most pronounced GSH-NS B and D cellular uptake. This difference in nanosponge cellular uptake in this cell line may be owing to differing uptake mechanisms, as cell surface thiols have been reported to affect disulfide-conjugated peptide cell entry [57]. Indeed, disulfide bridge cleavage may start at the cell surface via thiol/disulfide exchange reactions catalyzed by redox proteins such as thioredoxines [58]. Therefore, the 2D data on IC_50_ would appear to indicate that prostatic cancer cell lines are more sensitive to GSH-NS cytotoxic effects.

Worthy of note is that cell cycle analyses revealed a significant cell cycle arrest in the G_0_/G_1_ phase in all cell lines at 24 h IC_50_ values. Thus, to further investigate this cell cycle arrest, we analyzed a panel of genes that are involved in cell cycle regulation. Notably, the results show significant mRNA over-expression in the cell cycle progression regulators at G_1_, *CDKN1A*, and *CDKN2A*, which code for p21 and p16 that inhibit the cyclin-CDK2 and -CDK4 complexes, respectively. Apart from this, the mRNA expression of *CDC25A*, *CDK1*, *CDK2*, and *CDK4* was either unaffected or down-regulated in all cell lines. These data demonstrate that GSH-NS inhibition of cell proliferation is essentially owing to G_1_ cell cycle arrest, in agreement with previous reports by Choi et al. [59]. Interestingly, only HTC116 cells showed significant ROS production after GSH-NS exposure, which is most likely owing to their high GSH-NS cellular uptake.

Lastly, the investigation of nanosponges effects on MCS growth was carried out. The results were interesting as differences in the 2D study were observed. There were no significant differences between the two GSH-NS formulations in HCT116 and DU145 MCS, whereas there was a significant difference in the 2D HCT-116 culture. Indeed, IC_50_ values were significantly lower in the 2D cultures than in the 3D cultures, especially after 24 h incubation, where similar values were reached only after 72 h of incubation. For example, IC_50_ was twofold higher after 24 h in 3D cultures for HCT116 and three-fold higher in DU145 than in their respective monolayers. On the other hand, IC_1_ concentrations were significantly lower in HCT116 MCS than in HCT116 cell monolayers, whereas IC_1_ was quite similar both in DU145 spheroids and monolayers.

GSH-NS cytotoxicity might appear to be linked to disulfide-bond content in 2D cell monolayers as the formulation with the higher disulfide-bond content, GSH-NS D, had the lowest cytotoxic effect in all cell lines, except for the DU145 cell line. On the other hand, GSH-NS cytotoxicity was not influenced by the disulfide-bond content in MCS and the most pronounced cell growth decrease was observed in the colorectal cancer cell line, HCT116, after 24 h of exposure to GSH-NS. Tissue-like morphology and phenotypic change may be identified as the major factors in diminishing toxicity on MCS. This means that in vitro 3D cell culture models could act as an intermediate stage and bridge the gap between in vitro 2D and in vivo studies, which would extend current cellular level cytotoxicity to the tissue level and improve the predictive power of in vitro nanoparticle toxicology [60]. Finally, GSH-NSs showed a limited toxicity, leading to G_1_ cell cycle arrest, without membrane damage or oxidative stress generation at significantly higher concentrations about fifty times those used for the delivery of anticancer drugs.

## 4. Methods

### 4.1. Synthesis of Glutathione Responsive β-cyclodextrin-Based Nanosponges

Glutathione-responsive β-cyclodextrin-based nanosponges (GSH-NSs) were synthetized according to the method developed by Trotta et al. [35].

Briefly, GSH-NSs were obtained using a one-step synthetic route by reacting β-cyclodextrin and pyromellitic dianhydride, in the presence of 2-hydroxyethyl disulfide to insert disulfide bridges in the NS nanostructure [35]. Varying amounts of 2-hydroxyethyl disulfide were used to obtain a series of GSH-NS with different disulfide bridge percentages in the polymer matrix. In particular, 2-hydroxyethyl disulfide/β-cyclodextrin molar ratios of 1:20 and 1:5 were used for the synthesis of GSH-NS with low (GSH-NS B) and high disulfide-bridge content (GSH-NS D), respectively. The reaction was estimated at room temperature under stirring for 24 h. The nanosponges were then purified by Soxhlet extraction with acetone for a few hours. The percentage of sulfur in the two types of GSH-NS was measured by elemental analysis and was 0.62 and 1.90 for GSH-NS B and GSH-NS D, respectively.

### 4.2. Preparation of Glutathione Responsive β-cyclodextrin-Based Nanosponge Nanosuspension

GSH-NS nanosuspensions were prepared following the preparation protocol previously reported [35,38]. A weighed amount of GSH-NSs was suspended in a saline solution (NaCl 0.9%) at a concentration of 10 mg/mL. The suspension was homogenized by a high shear homogenizer (Ultraturrax^®^, IKA, Konigswinter, Germany) for 5 min at 24,000 rpm. The sample was then homogenized on a high-pressure homogenizer (EmulsiFlex C5, Avastin, Mannheim, Germany) for 90 min at a back pressure of 500 bar to further reduce the size of the nanosponges. The aqueous nanosponge nanosuspension was subsequently purified by dialysis (membrane cutoff 12,000 Da) to eliminate potential synthesis residues. The nanosuspension was stored at +4 °C and used for all experiments.

### 4.3. Preparation of Fluorescent Glutathione Responsive β-cyclodextrin-Based Nanosponges

Fluorescent GSH-responsive nanosponges were obtained by adding 6-coumarin (0.1 mg/mL) to the aforementioned aqueous GSH-NS nanosuspensions (previously described) (10 mg/mL) under stirring for 24 h at room temperature in the dark.

### 4.4. Characterization of Glutathione Responsive β-cyclodextrin-based Nanosponges

The two types of GSH-NS (GSH-NS B and D), either blank or 6-coumarin loaded, were characterized in vitro to measure their physico-chemical parameters. The average diameters, polydispersity indices, and zeta potential values were determined by photon correlation spectroscopy (PCS) and electrophoretic mobility using a 90 Plus Instrument (Brookhaven, NY, USA) at a fixed angle of 90° and a temperature of +25 °C. The analyses were performed on diluted GSH-NS samples (1:30 *v*/*v*). For zeta potential determination, the samples were placed in an electrophoretic cell where an electric field of approximately 15 V/cm was applied. Three batches were analyzed for each NS type and each measured value was the average of ten repetitions. Nanosponge morphology was evaluated by transmission electron microscopy (TEM) (Philips CM10 instrument, Eindhoven, Netherlands) after the diluted aqueous nanosponge nanosuspensions were sprayed onto a Form war-coated copper grid and air-dried.

### 4.5. Cell Culture and Treatment with Glutathione Responsive β-cyclodextrin-Based Nanosponges

Human colorectal cancer cell lines, HCT116 and HT-29 (ICLC, Interlab Cell Line Collection, Genova, Italy), and human prostatic carcinoma cell lines, DU145 and PC-3 (ICLC), were cultured in McCoy’s 5A Medium and RPMI-1640 Medium, respectively. These media were supplemented with 2 mM L-glutamine, 100 UI/mL penicillin, 100 µg/mL streptomycin, and 10% (*v*/*v*) heat-inactivated fetal calf serum (Sigma, ST Louis, MO, USA) in a humidified atmosphere of 5% CO_2_ air at 37 °C. At 85% confluence, cells were harvested with 0.25% trypsin and sub-cultured into 75 cm^2^ flasks, 6-well plates or 96-well plates according to need. Cells were allowed to attach to the surface for 24 h prior to treatment. GSH-NS B and D were then suspended in a cell culture medium and diluted to the appropriate concentrations. After treatment, the cells were harvested to determine cytotoxicity, cell cycle distribution and ROS production. The cells that were not exposed to GSH-NS were used as control conditions for each experiment.

### 4.6. Measurement of Basal Intracellular Reduced Glutathione Levels

The total glutathione level (GSSG + GSH) in HT-29, HCT116, DU154, and PC-3 cells were assayed by the Glutathione Assay Kit (Sigma, Milano, Italy), according to manufacturer’s instructions. The protein concentration (μg/mL) was quantified by the Qubit fluorometer (Invitrogen, Milan, Italy) and the Quant-IT Protein Assay Kit (Invitrogen, Milano, Italy). Calibration was performed by the application of a two-point standard curve, according to the manufacturer’s instructions.

Briefly, reduced glutathione (GSH) reacts with 5,5′-dithiobis(2-nitrobenzoic acid) (DTNB) in a recycling assay and produces glutathione disulfide (GSSG) and the 1,3,5-trinitrobenzene (TNB) anion, which can be detected by absorbance. In turn, the enzyme glutathione reductase then reduces GSSG, which release GSH that can react with another DTNB molecule. Therefore, the rate of TNB production is measured rather than a single determination of how much DTNB react with GSH, as it is proportional to the initial amount of GSH [61]. The plate was read at 412 nm on a microplate reader Asys UV 340 (Biochrom, Cambridge, UK) and the amount of GSH was expressed in nmol/µg protein.

### 4.7. Cell Proliferation Assay

The effect that GSH-NS B and D had on HCT116, HT-29, DU145, and PC-3 cell growth was evaluated by WST-1 cell proliferation assay (Roche Applied Science, Penzberg, Germany). Briefly, 2.0 × 10^3^ HT-29, 1.5 × 10^3^ HCT116, 5.0 × 10^2^ DU145, and 1.2 × 10^3^ PC-3 cells were seeded in 100 µL of growth medium in replicates (*n* = 8) in 96-well culture plates; the seeding density of each cell line was chosen according to the best proliferation rate. The medium was removed after 24 h and the cells were incubated with in an experimental medium containing differing GSH-NS B or GSH-NS D concentrations (0.5, 1.0, 2.0, and 3.0 mg/mL). At 24, 48, and 72 h, WST-1 reagent (10 µL) was added and the plates were incubated at 37 °C in 5% CO_2_ for 1.5 h. Well absorbance was measured at 450 and 620 nm (reference wavelength) on a microplate reader Asys UV 340.

Cell proliferation data were expressed as a percentage of control, that is, untreated cells. At 24, 48, and 72 h, the inhibition concentration 50% (IC_50_), defined as the dose of compound that inhibited 50% of cell growth, was interpolated from the growth curves, as was the inhibition concentration 1% (IC_1_), defined as the dose of compound that inhibited 1% of cell growth. Thus, to compare the effects of GSH-NS on the different cell lines, the IC_1_ and IC_50_ values obtained were used to carry out the following experiments.

### 4.8. Nanosponge Cellular Uptake Assays

Coumarin 6-loaded GSH-NS cellular uptake was assessed by cytofluorimetric analysis using a C6 flow cytometer (Accuri Cytometers, Ann Arbor, MI, USA) and imaging analysis using a DMI4000B fluorescence microscopy (Leica, Wetzlar, Germany). For flow cytometry analysis, 5.0 × 10^4^ cells were seeded in a six-well culture plate. Forty-eight hours after seeding, HT-29, HCT116, DU145, and PC-3 cells were treated with the respective not-cytotoxic (IC_1_) and cytotoxic (IC_50_) concentrations of either fluorescent GSH-NS B or fluorescent GSH-NS D at 24 h. After a 24 h incubation, the cells were washed three times with phosphate-buffered saline PBS, suspended in 250 µL PBS, and run on the flow cytometer with 488 nm excitation. Intracellular fluorescence was expressed as integrated mean fluorescence intensity (iMFI), which was the product of the frequency of 6-coumarin-loaded GSH-NS positive cells and the mean fluorescence intensity.

Microscopy observation was carried out after glass coverslips were placed in 24-well plates and the cells seeded at a density of 5.0 × 10^4^ cells/coverslip for 48 h of incubation. The coumarin 6-loaded nanosponges were then added at the respective IC_50_ values for GSH-NS B and D, and incubated for 24 h. The cells were incubated with 1 µg/mL of 4′,6-diamidino-2-phenylindole (DAPI) for nuclear counterstaining 30 min before the programmed stop time. After the cells were washed with PBS, the cells on the coverslip were mounted on a glass slide, observed under a fluorescence microscope, and photographed.

### 4.9. Lactate Dehydrogenase Leakage Assay

Lactate dehydrogenase (LDH) is an enzyme that is widely present in cytosol and catalyzes the conversion of lactate to pyruvic acid. If plasma membrane integrity is disrupted, the LDH leaks into culture media, increasing its extracellular level, and the amount of LDH release is proportional to the number of damaged cells [62]. The LDH leakage was evaluated by the LDH-Cytotoxicity Detection Kit (Roche Diagnostic, Indianapolis, USA), according to manufacturer’s instructions. Briefly, 96-well plates were seeded with HT-29, HCT116, DU145, and PC-3 cell lines at a density of 2.0 × 10^3^, 1.5 × 10^3^, 5.0 × 10^2^, and 1.2 × 10^3^ cells/100 µL culture medium, respectively. Twenty-four hours after the seeding, 100 µL of different concentrations (0.5, 1.0, 2.0, and 3.0 mg/mL) of GSH-NS B or GSH-NS D was added to the wells. The plates were then incubated for 24, 48, and 72 h, at 37 °C, in a humidified atmosphere of 5% CO_2_ air. Cell-free culture media were then collected and incubated with the same volume of reaction mixture for 30 min. LDH activity was measured at 490 nm on a microplate reader Asys UV 340. The background control was obtained by measuring the LDH activity of the assay medium, the untreated control by measuring the LDH activity of untreated cells, and the positive control by measuring the maximum releasable LDH activity after the treatment with the lysis buffer. The LDH leakage percentage was calculated as follows: LDH leakage (%) = (experimental value−untreated control)/(positive control−untreated control) × 100, and is the mean of three independent wells.

### 4.10. Cell Cycle Analysis

Cell cycle distribution was evaluated 24 h after cell treatment with the respective IC_50_ of GSH-NS B or GSH-NS D. The occurrence of the so-called sub-G_0_/G_1_ peak, which is a distinct cell population characterized by subdiploid DNA fluorescence and might correlate with the internucleosomal DNA fragmentation typical of apoptosis (Pozarowski and Darzynkiewicz, 2004), was also evaluated. Briefly, 1 × 10^6^ HCT116, 1 × 10^6^ HT-29, 1 × 10^6^ DU145, and 1 × 10^6^ PC-3 cells were incubated with 2 µM of the live cell staining Vybrant Dye Cycle Green (Invitrogen) for 30 min at 37 °C. The samples were run on a flow cytometer with 488 nm excitation to measure Vybrant Dye Cycle Green staining and data analysis was performed by FCS Express software version 4 (BD Bioscience, Milano, Italy).

### 4.11. Real Time Reverse Transcriptase-Polymerase Chain Reaction (RT-PCR)

Total RNA was isolated from the HCT116, HT-29, DU145, and PC-3 cells, 24 h after incubation with the respective GSH-NS B or GSH-NS D IC_50_. Briefly, the cells were collected in RNA Cell Protection Reagent (Qiagen, Milano, Italy) and stored at −80 °C. Total RNA was obtained by the RNeasy Plus Mini Kit (Qiagen Milano, Italy). Total RNA concentration (µg/mL) was determined using the fluorometer Qubit (Invitrogen) and the Quant-IT RNA Assay Kit (Invitrogen). Calibration was carried out by applying a two points standard curve, according to the manufacturer’s instructions. RNA sample integrity was determined by the Total RNA 6000 Nano Kit (Agilent Technologies, Milano, Italy) using the Agilent 2100 Bioanalyzer (Agilent Technologies, Milano, Italy).

Real-time RT-PCR analysis was carried out using 1 µg of total RNA, which was reverse transcribed in a 20 µL cDNA reaction volume using the QuantiTect Reverse Transcription Kit (Qiagen, Milano, Italy). Each 10 µL real-time RT-PCR reaction was obtained using 12.5 ng of cDNA, according to the manufacturer’s instructions. Quantitative RT-PCR was performed by the SsoFast EvaGreen (Bio-Rad, Milan, Italy) and the QuantiTect Primer Assay (Qiagen, Milano, Italy) was used as the gene-specific primer pair for the studied gene panel (Table 4).

The transcript of the reference gene 18S ribosomal RNA (*RRN18S*) was used to normalize mRNA data and real-time RT-PCR was performed by the MiniOpticon Real Time PCR system (Bio-Rad, Milan, Italy). The PCR protocol conditions were as follows: a HotStarTaq DNA polymerase activation step at +95 °C for 30 s, followed by 40 cycles at +95 °C for 5 s and +55 °C for 10 s. All runs were performed on at least three independent cDNA preparations per sample and all samples were run in duplicate. At least two non-template controls were included in each PCR run. Quantification data analyses were performed by the Bio-Rad CFX Manager software version 1.6 (Bio-Rad, Milan, Italy), according to the manufacturer’s instructions. These analyses were performed in compliance with MIQE guidelines (Minimum Information for Publication of Quantitative Real-time PCR Experiments) [63].

### 4.12. Reactive Oxygen Species Production Assay

The production of intracellular reactive oxygen species (ROS) was measured by flow cytometry using dichlorofluorescein-diacetate (DCFH-DA) (Sigma, Milano, Italy) as the oxidation-sensitive probe. Briefly, after 1, 12, and 24 h cell exposure to the respective GSH-NS B or GSH-NS D IC_1_ and IC_50_ at 24 h, HT-29, HCT116, DU145, and PC-3 cells were washed twice with PBS in six-well plates and incubated with 10 µM DCFH at 37 °C in the dark for 30 min. The cells were then washed with PBS, trypsinized, collected in 500 µL of PBS, and analyzed. ROS production was expressed as iMFI ratio, that is, the difference between the iMFI of treated and untreated cells over the iMFI of untreated cells (iMFI is the product of the frequency of ROS-producing cells and the median fluorescence intensity).

### 4.13. Cell Growth and Nanosponge Cellular Uptake Assays on Three-Dimensional Cell Culture

Cell suspensions (250-cell spheroids) 40 µL were dispensed into the access hole at each cell culture site to form a hanging drop on a Perfecta3D^®^ 96-well Hanging Drop Plate (3D Biomatrix, Ann Arbor, MI, USA). On day 8 of the HCT116 and DU145 spheroid culture, 15 µL of different GSH-NS B or GSH-NS D concentrations (0.5, 2.0, 4.0, and 6.0 mg/mL) was added to each cell hanging drop and MCS growth was analyzed at 24, 48, and 72 h after nanosponge incubation. Noteworthy is the fact that we had to use a different concentration range for 3D cell growth assay (0.5, 2.0, 4.0, and 6.0 mg/mL) to obtain the dose-response data necessary to calculate the IC_50_ values from the one used in the 2D cell growth assay (0.5, 2.0, 4.0, and 3.0 mg/mL). Phase contrast photographs were taken by the DMI4000B microscope (Leica, Milano, Italy) and the diameter of each MCS was measured by Leica Application Suite Software (Leica) and the volume (V) was calculated using the equation V = 4/3πr^3^. Coumarin 6-loaded nanosponge uptake by MCS at the respective IC_50_ at 24 h for GSH-NS B or GSH-NS D was analyzed by fluorescence microscopy using a DMI4000B microscope (Leica).

### 4.14. Statistical Analysis

The results are expressed as the average value ± standard deviation (St.Dev) of three independent experiments. Median-effect analysis was performed by CalcuSyn software version 2.11 (Biosoft, Cambridge, UK) to calculate the values of the concentration required to cause a 1% inhibition of cell growth (IC_1_) and for a 50% inhibition of cell growth (IC_50_) for each nanosponge formulation. Statistical analyses were performed on Prism software version 6 (Graph-Pad, La Jolla, CA, USA) using a Student’s *t*-test and one-way analysis of variance (ANOVA) to calculate the threshold of significance as appropriate. Statistical significance was set at *p* < 0.05.

## Figures and Tables

**Table 1:** The physico-chemical characteristics of glutathione-responsive β-cyclodextrin-based nanosponge (GSH-NS) formulations.

	Average Diameter ± SD (nm)	Polydispersity Index (PDI)	Zeta Potential ± SD (mV)
**Blank GSH-NS B**	183.4 ± 15.3	0.23 ± 0.02	−31.58 ± 3.82
**Blank GSH-NS D**	180.5 ± 6.7	0.21 ± 0.01	−31.24 ± 3.05
**Fluorescent GSH-NS B**	188.3 ± 10.2	0.22 ± 0.01	−29.98 ± 2.74
**Fluorescent GSH-NS D**	185.9 ± 12.5	0.22 ± 0.02	−30.55 ± 2.66

**Table 2:** Glutathione-responsive β-cyclodextrin-based nanosponge (GSH-NS) inhibition concentration (IC) values on 2D cell cultures.

**HCT116 IC Values (mg/mL ± St.Dev) on 2D Cultures**								
**24 h**	**GSH-NS** **B**	**GSH-NS** **D**	**48 h**	**GSH-NS** **B**	**GSH-NS** **D**	**72 h**	**GSH-NS B**	**GSH-NS** **D**
IC_1_	0.27 ± 0.03	0.51 ± 0.04	IC_1_	0.47 ± 0.03	0.65 ± 0.03	IC_1_	0.50 ± 0.04	0.66 ± 0.05
IC_50_	1.86 ± 0.31	2.62 ± 0.39 *	IC_50_	1.60 ± 0.27	2.35 ± 0.40 *	IC_50_	1.54 ± 0.19	2.38 ± 0.41 *
**HT-29 IC Values (mg/mL ± St.Dev) on 2D Cultures**								
**24 h**	**GSH-NS B**	**GSH-NS D**	**48 h**	**GSH-NS** **B**	**GSH-NS D**	**72 h**	**GSH-NS** **B**	**GSH-NS** **D**
IC_1_	0.05 ± 0.00	0.05 ± 0.00	IC_1_	0.15 ± 0.01	0.65 ± 0.03 *	IC_1_	0.27 ± 0.02	0.65 ± 0.03 *
IC_50_	1.71 ± 0.25	2.76 ± 0.35 *	IC_50_	1.58 ± 0.21	2.80 ± 0.45 *	IC_50_	1.37 ± 0.30	2.39 ± 0.31 *
**DU145 IC Values (mg/mL ± St.Dev) on 2D Cultures**								
**24 h**	**GSH-NS B**	**GSH-NS D**	**48 h**	**GSH-NS** **B**	**GSH-NS D**	**72 h**	**GSH-NS** **B**	**GSH-NS** **D**
IC_1_	0.01 ± 0.00	0.05 ± 0.00	IC_1_	0.05 ± 0.00	0.03 ± 0.00	IC_1_	0.17 ± 0.01	0.12 ± 0.01
IC_50_	1.85 ± 0.40	2.34 ± 0.36	IC_50_	1.37 ± 0.28	1.57 ± 0.32	IC_50_	1.38 ± 0.25	1.56 ± 0.30
**PC-3 IC Values (mg/mL ± St.Dev) on 2D Cultures**								
**24 h**	**GSH-NS B**	**GSH-NS D**	**48 h**	**GSH-NS** **B**	**GSH-NS** **D**	**72 h**	**GSH-NS** **B**	**GSH-NS** **D**
IC_1_	0.04 ± 0.00	0.05 ± 0.00	IC_1_	0.06 ± 0.01	0.43 ± 0.03 *	IC_1_	0.35 ± 0.02	0.43 ± 0.03
IC_50_	1.65 ± 0.27	2.56 ± 0.38 *	IC_50_	1.20 ± 0.22	2.09 ± 0.25 *	IC_50_	1.37 ± 0.31	1.68 ± 0.27

Statistically significant difference between the two nanosponge formulations: * *p* < 0.05.

**Table 3:** GSH-NS IC values in the 3D cell cultures.

**HCT116 IC Values (mg/mL ± St.Dev) on 3D Cultures**								
**24 h**	**GSH-NS B**	**GSH-NS D**	**48 h**	**GSH-NS B**	**GSH-NS D**	**72 h**	**GSH-NS B**	**GSH-NS D**
IC_1_	0.01 ± 0.00	0.02 ± 0.00	IC_1_	0.01 ± 0.00	0.01 ± 0.00	IC_1_	0.01 ± 0.00	0.01 ± 0.00
IC_50_	3.92 ± 0.95	4.19 ± 0.98	IC_50_	2.75 ± 0.18	3.05 ± 0.31	IC_50_	1.98 ± 0.15	2.22 ± 0.21
**DU145 IC Values (mg/mL ± St.Dev) on 3D Cultures**								
**24 h**	**GSH-NS B**	**GSH-NS D**	**48 h**	**GSH-NS B**	**GSH-NS D**	**72 h**	**GSH-NS B**	**GSH-NS D**
IC_1_	0.02 ± 0.00	0.04 ± 0.00	IC_1_	0.03 ± 0.00	0.02 ± 0.00	IC_1_	0.07 ± 0.01	0.02 ± 0.00
IC_50_	5.38 ± 1.15	5.80 ± 1.78	IC_50_	2.68 ± 0.25	3.24 ± 0.34	IC_50_	2.01 ± 0.24	2.26 ± 0.23

Statistically significant difference between the two nanosponge formulations: ns.

**Table 4:** Gene description.

Gene	Primer Codes	Description
*CDC25A*	QT00001078	cell division cycle 25 homolog A
*CDK1*	QT00042672	cyclin-dependent kinase 1
*CDK2*	QT00005586	cyclin-dependent kinase 2
*CDK4*	QT00016107	cyclin-dependent kinase 4
*CDKN1A*	QT00031192	cyclin-dependent kinase inhibitor 1A, p21
*CDKN2A*	QT00089964	cyclin-dependent kinase inhibitor 2A, p16
*RRN18S*	QT00199367	18S ribosomal RNA
